# Usage and Longitudinal Effectiveness of a Web-Based Self-Help Cognitive Behavioral Therapy Program for Panic Disorder

**DOI:** 10.2196/jmir.7.1.e7

**Published:** 2005-03-26

**Authors:** Peter Farvolden, Eilenna Denisoff, Peter Selby, R Michael Bagby, Laura Rudy

**Affiliations:** ^1^Centre for Addiction and Mental HealthToronto ONCanada

**Keywords:** Anxiety, depression, disorders, cognitive behavioural therapy, CBT, self-help, Web-based, treatment, primary care, collaborative, management, access, mental health

## Abstract

**Background:**

Anxiety disorders are common problems that result in enormous suffering and economic costs. The efficacy of Web-based self-help approaches for anxiety disorders has been demonstrated in a number of controlled trials. However, there is little data regarding the patterns of use and effectiveness of freely available Web-based interventions outside the context of controlled trials.

**Objective:**

To examine the use and longitudinal effectiveness of a freely available, 12-session, Web-based, cognitive behavioral therapy (CBT) program for panic disorder and agoraphobia.

**Methods:**

Cumulative anonymous data were analyzed from 99695 users of the Panic Center. Usage statistics for the website were examined and a longitudinal survey of self-reported symptoms for people who registered for the CBT program was conducted. The primary outcome measures were self-reported panic-attack frequency and severity at the beginning of each session (sessions 2-12).

**Results:**

Between September 1, 2002 and February 1, 2004, there were 484695 visits and 1148097 page views from 99695 users to the Panic Center. In that same time period, 1161 users registered for the CBT program. There was an extremely high attrition rate with only 12 (1.03%) out of 1161 of registered users completing the 12-week program. However, even for those who remained in the program less than 12 weeks we found statistically significant reductions (*P*<.002) in self-reported panic attack frequency and severity, comparing 2 weeks of data against data after 3, 6, or 8 weeks. For example, the 152 users completing only 3 sessions of the program reduced their average number of attacks per day from 1.03 (week 2) to 0.63 (week 3) (P<.001).

**Conclusions:**

Freely available Web-based self-help will likely be associated with high attrition. However, for the highly self-selected group who stayed in the program, significant improvements were observed.

## Introduction

Anxiety disorders are common problems that result in enormous suffering and economic costs [1]. Unfortunately, a large proportion of people who suffer from anxiety disorder remain either untreated or inadequately treated [2,3]. Effective treatments for anxiety disorders include pharmacological as well as psychotherapeutic approaches and the majority of patients with anxiety disorders respond to appropriate treatment. However, limited access to evidence-based psychotherapy outside of specialized clinics and research settings often renders pharmacotherapy the most practical first-line treatment option in primary care [4-7].

Self-help therapy for anxiety disorders has been found to be effective, especially when the interventions are tailored to the individual's specific symptoms and situation and administered with a minimal amount of professional guidance and support [6-10]. Web-based self-help is likely to be more effective than traditional bibliotherapy, insofar as it has the potential to be interactive, tailored to an individual's specific needs, able to monitor progress and offer peer support, and augment the traditional physician-patient relationship [7,10-12].

There has been some research on Web-based programs designed to provide relatively generic CBT interventions for depression and anxiety [13], programs designed specifically to provide self-guided CBT for depression [14-16], and programs for anxiety disorders [17-20] and especially panic disorder [21-26]. Most recently, Carlbring and colleagues [27] have reported that Web-based self-help plus minimal therapist contact can be equally as effective as traditional therapist administered CBT in the treatment of panic disorder.

Although the evidence for the efficacy of Web-based self-help for mood and anxiety disorders from controlled trials is encouraging, it is important to determine how such programs are utilized and to estimate their effectiveness when accessed by diverse, less well-selected groups of users under less controlled conditions. To this end, Christensen et al [28] recently reported the results of study in which they compared changes in anxiety and depression symptoms of spontaneous users and trial participants of a CBT website. Christensen et al [28] reported that public registrants did not differ from trial participants in baseline measures including gender, age, and initial level of depression. Most importantly, both groups improved across the training program, although only 15.6% of public registrants completed the program. While such data suggest that public registrants to a cognitive behavior therapy website can experience as much improvement in symptoms as participants in a controlled trial, there is very little data on the patterns of use and effectiveness of Web-based interventions specifically for panic disorder outside of the context of controlled trials.

In contrast to previous reports of the efficacy of computer-assisted and Web-based interventions for anxiety in well-controlled research settings, in the present study we examined the use and effectiveness in an uncontrolled visitor population of a freely available Web-based CBT program for panic disorder.

## Method

### Description of the Intervention

The Panic Center [29] is an interactive website dedicated to helping those who suffer from panic disorder and agoraphobia. The goal is to promote interaction between people who suffer from panic disorder and their health care professionals. People who visit the Panic Center are a self-selected sample of people who choose to use the Internet to access information and to seek self-help for panic disorder and agoraphobia. Features (tools) of the Panic Center include educational content, a moderated support group, a validated screening test for mood and anxiety disorders [30], a panic symptom diary, and a 12-session self-help CBT program (the Panic Program). Visitors to the Panic Center can use any one of the individual tools either on their own or in collaboration with a health care professional. However, the components of the Panic Program include a combination of the tools described above designed to provide a comprehensive program for the assessment, treatment and maintenance of improvement of the symptoms of panic disorder and agoraphobia.

**Figure 1:**
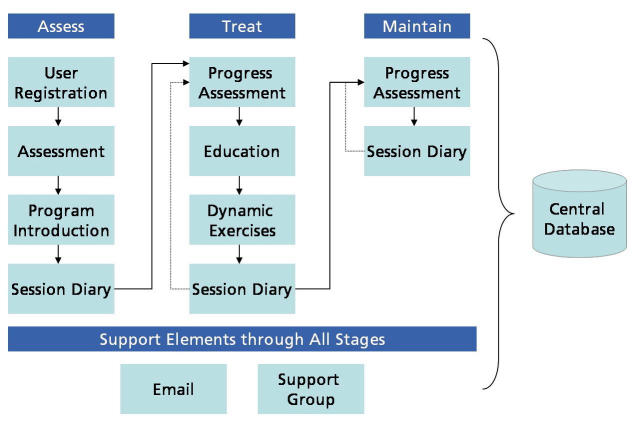
Panic program process

**Figure 2:**
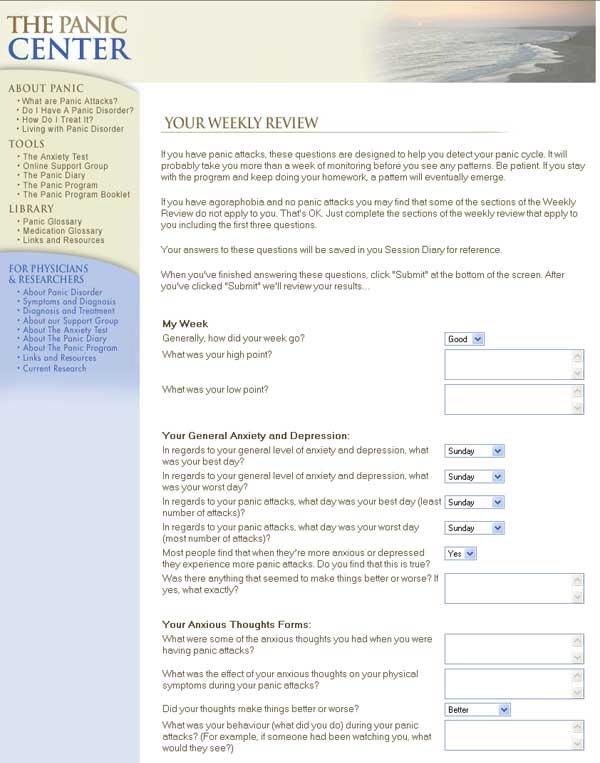
Sample weekly review at session 2

As illustrated in Figure 1, following registration for the Panic Program, users complete an assessment of their current symptoms of anxiety and depression using a screening questionnaire (Web-Based Depression and Anxiety Test, WB-DAT, see below). Following the initial assessment, users are free to proceed through the Web-based 12-session CBT program at their own pace. The sessions are designed to be completed in weekly intervals, hence completion of the entire program normally takes 12 weeks. In order to register for the program users are asked to provide an anonymous email address, select a screen name that is different from their own, provide basic demographic data (age, gender and country of residence) and provide preliminary information on their panic symptoms (Multimedia Appendix Slide 2). Users who register for the Panic Program are automatically registered to use the panic symptom diary. At the beginning of each session users complete a Weekly Review (Progress Assessment) Figure 2 in which they respond to a variety of questions about their current symptoms and assigned homework. The results of these assessments, as well as the results of dynamic exercises completed during each session, are saved to the user's Session Diary (Multimedia Appendix Slide 4). As part of the CBT, each session provides educational text and suggests exercises (Multimedia Appendix Slide 5). Finally, following the completion of session 12, users are asked to respond to a number of specific questions about their current symptoms and symptom improvement as well as a second screening assessment of their symptoms of anxiety and depression (Multimedia Appendix Slides 6 and 7). Following completion of the 12-session program, users can continue to use the Session Diary and panic symptom diary indefinitely to continue to improve and maintain their gains. Users of the CBT program have indefinite access to the moderated support group (Multimedia Appendix Slide 8) as well as individualized email support and advice.

As an alternative to using the Web-based treatment program, users can download an Adobe version of the 12-session program and use the hard copy as a traditional self-help book. Although this option reduces the number of people using the Web-based program and options for collecting data about the use and effectiveness of the program, it is offered in the interest in maximizing the dissemination and use of the program.

The following describes some of the components in more detail.

#### Support Group and Email

The support group format consists of asynchronous communication (bulletin board format) between members of the support community and the moderators. Users of the support group also have access to individualized email support and advice from the moderators, who are Registered Nurses (Multimedia Appendix Slide 8).

#### Screening Assessment (WB-DAT)

**Figure 3:**
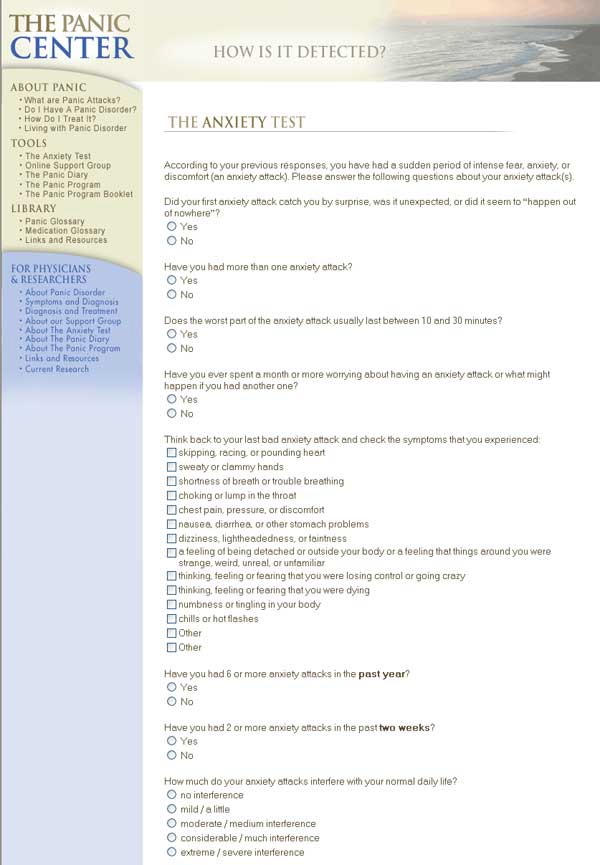
WB-DAT panic disorder screener

The Web-Based Depression and Anxiety Test (WB-DAT) is a self-report screening tool for mood and anxiety disorders compatible with the DSM-IV [31] and the *International Classification of Diseases and Related Health Problems*, tenth revision (ICD-10) [32] diagnostic systems. Preliminary data suggest that the WB-DAT is reliable for identifying patients with and without major depressive disorder (MDD) and the anxiety disorders (Figure 3).

#### Symptom Diary

**Figure 4:**
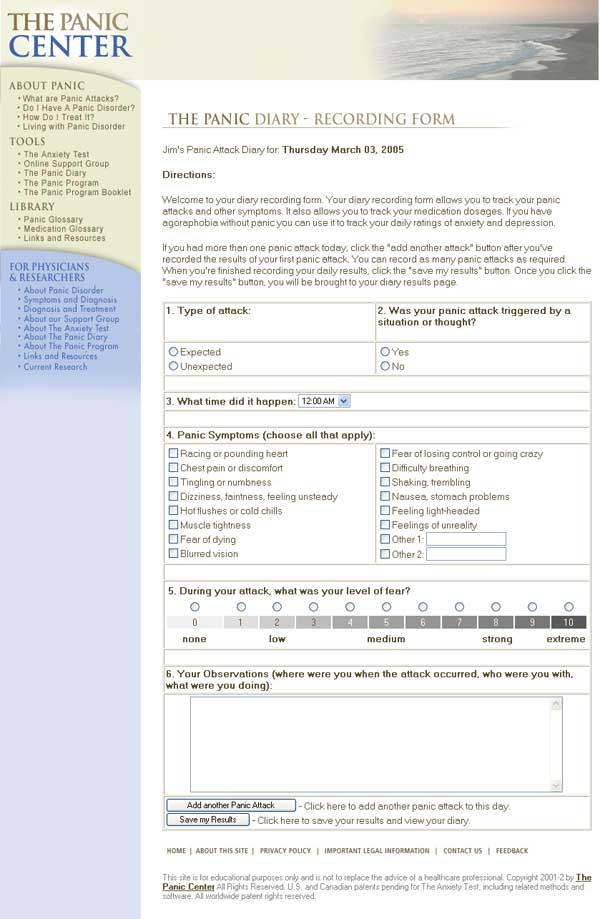
Panic diary recording form

The panic symptom diary (Panic Diary) allows users to record and track the frequency and severity of their panic attacks, their overall daily level of anxiety and depression, and their medication(s) and dose(s) Figure 4. A graphics interface allows users to track their symptoms over time.

#### CBT Program

The CBT program was designed based on current evidence for the effective components of CBT interventions for panic disorder and agoraphobia. The essential components of the CBT program include orientation to the cognitive behavioural model of panic disorder and agoraphobia, goal setting, exposure work exercises, cognitive restructuring, interoceptive exposure work, relaxation training, and information about lifestyle change and stress management (Multimedia Appendix Slide 5). Users are assigned homework to complete each week. As mentioned previously, users are at the beginning of each session asked to respond to a number of questions about their symptoms, homework and progress to date (Weekly Review, see Figure 2. These results as well as the results from the dynamic exercises completed during each session are stored in the user's Session Diary and can be viewed by the user at any time.

### Data Collection

In order to determine the overall usage of the individual Panic Center tools, we examined log statistics regarding website usage and traffic, including overall statistics regarding the number of visitors to the website, page views, and usage of the screening test, symptom diary and support group. With respect to evaluating the effectiveness of the CBT program, we conducted a longitudinal survey examining data from the Weekly Review questions as well as the screening assessments conducted at registration and at the end of session 12.

#### Ethics and Privacy

This study was approved by the Research Ethics Board at the Centre for Addiction and Mental Health in Toronto, Ontario, in accordance with all applicable regulations. With respect to the log statistics, the number of unique visitors was determined based on IP addresses. WebTrends was used to analyze log files. No techniques were employed to analyze the log file for identification of multiple entries. With respect to the informed consent process for evaluating the use of the panic symptom diary and the WB-DAT, and effectiveness of the CBT program, users were informed of the approximate length of time of the surveys, which data are stored and where and for how long. Users were neither informed of the specific name of the investigators nor the specific purpose of the study. They were informed that “. . . anydata that is collected is cumulative. That means we compile your data with the results of others. We do not keep individual statistics and we are unable to find out who you are.” The policy also informed users that “Your information will be grouped with other peoples' information so that independent researchers can conduct research to improve the system for other people with panic disorder and agoraphobia. We will not sell e-mail identification, names or addresses to third parties.”

No personal identifying information was collected or stored. A number of specific measures were taken to protect the privacy of the participants and unauthorized access including the following:

1. Users do not have to provide any identifying information when they access the website or register to use any of the tools. Therefore, these are essentially cumulative and anonymous survey data.
2. Users are not required to provide any identifying information when they register for the WB-DAT, support group, symptom diary, or CBT program. In order to ensure anonymity, they are in fact discouraged from using their real names or email addresses. Users are explicitly asked to use a pseudonym when they use the program and are asked to create a hotmail or Yahoo account using a pseudonym so that they cannot be identified by their email address.
3. The design of the Panic Center strictly adheres to international laws that protect privacy. The data collection methodologies follow guidelines set forth by the Personal Information Protection and Electronic Documents Act (PIPEDA) [33], the Health Insurance Portability and Accountability Act of 1996 (HIPPA) [34] and Directive on Privacy and Electronic Communications – European Union (Directive 2002/58/EC) [35].
4. Security of the database is assured by a robust firewall setup that sits at the edge of their Web network to secure the flow of data. The network operations are manned 24 hours a day, 7 days a week, and a security officer is present round-the-clock. Closed Circuit Television (CCTV) monitors all access points at the server co-location facility, Peer1 Networks [36].

#### Electronic Surveys

The usability and technical functionality of the electronic data collection was rigorously tested and subjected to quality assurance (tested on multiple browsers, error checking code implemented, unit testing) before data collection began. The format of data collection was a “closed survey” posted on a website and initial contact was made on the Internet. No incentives were offered. Items were not randomized. The maximum number of items per page was 32. A completeness check was performed using JAVAscripterror checking. All questions were static and mandatory. Adaptive questioning was not used. Most questions did not allow for a not applicable response. Respondents were not able to review and change their responses. The “view rate” (as defined by Eysenbach [37]) for the first session of the CBT program was 1161 out of 99695 or 1.16%. The “completion rate” [37] for the CBT program was 12 out of 1161 or 1.03%. Duplicate entries were prevented by ensuring that the survey was only displayed once to each user. No cookies or time stamps were used. Each user who registered had a unique email address as the “primary key” to identify them as a unique user. Data from all users who registered for the program were analyzed. No statistical methods were used to adjust for a nonrepresentative sample. Data were stored in a SQL database and analyzed using SPSS.

### Participants

The sample was a self-selected convenience sample. Cumulative anonymous logfile data were analyzed from 99695 users of the Panic Center from September 1, 2002 to February 1, 2004. In addition, we examined self-reported outcome data from 1161 people who registered for the CBT program (the Panic Program) within the same time frame.

### Measures

#### Log Statistics Regarding Website Usage and Traffic

We examined cumulative data regarding website usage (traffic) including number of visits, number of page views, number of unique visitors, and average viewing time (length of visit).

#### Usage of the Screening Measure, Panic Symptom Diary, and Support Group

We examined cumulative data regarding usage of the WB-DAT including number of tests completed, number of males and females completing the test, average number of diagnoses per user and the relative frequency for users meeting screening criteria for the anxiety disorders, major depressive disorder (MDD), and dysthymia. In addition, we asked users what they intended to do with their screening test results. We examined cumulative data regarding use of the symptom diary including number of registered users and their gender. We examined cumulative data regarding usage of the support group including number of visitors, number of registered members, and number of posts.

#### Usage and Longitudinal Survey of Effectiveness of the CBT Program

When individuals registered for the Panic Program, they were asked a number of questions about their current symptoms, including questions about the frequency and intensity of their panic attacks, as well as the degree to which their symptoms interfered with their daily lives. In addition, users were asked to indicate whether they were using the program on their own or in collaboration with a health care professional. At the beginning of each session, users were asked a number of questions regarding their symptoms, homework and progress to date (Weekly Review). At the end of session 12 users were asked to respond to a number of questions regarding the frequency and severity of their panic attacks as well as the degree to which their symptoms interfered with their daily lives. Finally, users are asked to complete the WB-DAT at the time they register for the program as well as at the end of session 12.

We evaluated the effectiveness of the Panic Program in three ways. First we used the Weekly Review data to compare the reported frequency and severity of panic attacks at the beginning of sessions 2, 4, 6, 8, 10, and 12. Second, we compared data on the degree to which users' panic attacks interfered with their daily lives at the time they registered for the program and at the end of session 12. Third, we compared users' WB-DAT data at registration and at the end of session 12 to determine the number of users who met screening criteria for DSM-IV Axis I diagnoses at the time they registered for the program compared to the end of session 12. Dimensional data regarding frequency and severity of panic attacks and interference in daily life were analyzed using paired-samples *t* tests.

## Results

### Log Statistics Regarding Website Use and Traffic

Between September 1, 2002 and February 1, 2004, there were 484695 visits and 1148097 page views from 99695 unique visitors to the Panic Center. The average length of a visit was 13 minutes and 11 seconds (SD [standard deviation] 4 minutes, 21 seconds). There were 28123 unique visitors to the Panic Program, WB-DAT, and Panic Diary and 356134 page views of those features.

### Use of the Screening Test, Panic Symptom Diary, and Support Group

Between September 1, 2002 and February 1, 2004, 15269 users completed the WB-DAT. Table 1 describes the number of tests completed (male/female), as well as the number of users who met screening criteria for 0-8 disorders. Table 2 describes the number of users who met screening criteria for each of the DSM-IV disorders screened for by the WB-DAT.

**Table 1:** Number of screening diagnoses criteria met by users of the WB-DAT

**Users**	**Total**	**% (N=15269)**
Total males	5075	33.24
Total females	10194	66.76
Total tests with no diagnosis	1933	12.66
Total tests with 1 diagnosis	3691	24.17
Total tests with 2 diagnoses	2731	17.89
Total tests with 3 diagnoses	2237	14.65
Total tests with 4 diagnoses	1890	12.38
Total tests with 5 diagnoses	1474	9.65
Total tests with 6 diagnoses	1056	6.92
Total tests with 7 diagnoses	257	1.68
Total tests with 8 diagnoses	0	0

**Table 2:** Number of users meeting screening criteria on the WB-DAT

**Screening Diagnosis**	**Total**	**% (N=15269)**
No diagnosis	1933	12.66
Major depressive disorder	2021	13.24
Dysthymic disorder	4107	26.90
Generalized anxiety disorder	5891	38.38
Obsessive compulsive disorder	2504	16.40
Panic disorder with agoraphobia	4360	28.55
Panic disorder without agoraphobia	254	1.66
Agoraphobia without a history of panic disorder	2971	19.46
Social phobia (generalized subtype)	3643	23.86
Social phobia (nongeneralized subtype: public speaking)	3525	23.09
Specific phobia	40	00.26
Post-traumatic stress disorder	3707	24.28
Acute stress disorder	44	00.29

Out of 15229 users, 6687 (43.79%) responded to the survey. Of these 1388 (20.76%) reported that they intended to share the results with their doctor; 2517 (37.64%) reported that they were going to think about sharing the results with their doctor; 777 (11.62%) reported that they were not going to share the results with their doctor; 229 (3.42%) reported that they were health care professionals reviewing the test; and 1776 (26.56%) had “no comment.” Of the total number of users who completed the screening test, 4003 (26.21%) printed their results (Final Report), 1676 (10.97%) emailed their results to themselves, and 198 (1.29%) emailed their results to a health care professional.

Between September 1, 2002 and February 1, 2004, 493 (357 [72.41%] female and 136 [27.59%] male) users registered to use the panic symptom diary (Panic Diary) without also registering for the CBT program. During the same time period, 1451 users registered for the online support group and there were a total of 6664 posts and 75622 visitors. On average, each post was viewed by 8.81 (SD 2.34) visitors.

### Use and Longitudinal Survey of Effectiveness of the CBT Program

Between September 1, 2002 and February 1, 2004, 856 (73.90%) females and 305 (26.1%) males registered for the Panic Program. Out of 1161, 126 (11%) reported that they were using the program “with a health care professional” and 1065 (92%) reported that they were using it “on their own.” In addition, 190 users reported that they were “a health care professional reviewing the program.” Their data were excluded from further analyses. The Panic Program in booklet form was downloaded by 1059 users. Table 3 presents the number of users who completed each session of the 12-session CBT Program, showing a substantial degree of attrition from session to session, with only 12 out of 1161 original users remaining at the end of the program.

**Table 3:** Number of users who completed each session of the 12-session CBT program

**Session**	**Completers**	**% Users from Previous Session**
Session 1	1161	N/A
Session 2	525	45.22
Session 3	152	28.95
Session 4	145	95.39
Session 5	91	62.76
Session 6	46	50.55
Session 7	39	84.78
Session 8	30	76.92
Session 9	28	93.33
Session 10	22	78.57
Session 11	16	72.72
Session 12	12	75.00

The primary outcome measure for the effectiveness of the Panic Program was user's self-report of panic attack frequency and severity at the beginning of each session (sessions 2-12). At the beginning of each session users were asked to report the number of panic attacks they had experienced per day for the previous week and the average intensity of those panic attacks on a scale from 0 to 10 with 0 being “no panic” and 10 being as intense as the “worst attack ever” Figure 2. Results of paired-sample *t* tests for these variables are presented in Tables 4 and 5. There were statistically significant reductions in panic attack frequency and severity across treatment, including significant reductions between sessions 2 and 3 (*P*<.001).

**Table 4:** Average number of panic attacks per day in the past week

**Interval**	**Average # of Attacks/Day (SD)**	***df*****	**t**	***P* (2-tailed)**
Week 2 Week 3 (n=152)	1.03 (1.47) 0.63 (1.04)	1.151	3.983	<.001
Week 2 Week 6 (n=46)	1.04 (1.38) 0.30 (0.76)	1.45	3.995	<.001
Week 2 Week 8 (n=30)	1.07 (1.41) 0.37 (0.85)	1.29	3.427	.002
Week 2 Week 12 (n=12)	1.00 (1.60) 0.08 (0.29)	1.11	2.303	.042

**Table 5:** Average intensity of panic attacks in the past week

**Interval**	**Average Intensity of Attacks (SD)**	***df*****	**t**	***P* (2-tailed)**
Week 2 Week 3 (n=152)	3.63 (3.17) 2.50 (2.34)	1.151	4.512	< .001
Week 2 Week 6 (n=46)	3.30 (3.16) 0.96 (2.39)	1.45	5.580	< .001
Week 2 Week 8 (n=30)	3.10 (3.19) 1.07 (2.48)	1.29	4.210	< .001
Week 2 Week 12 (n=12)	2.08 (2.81) 0.33 (1.16)	1.11	2.303	.044

Only 12 users completed all outcome measures, including the WB-DAT. At session 1, those 12 individuals met criteria for an average of 1.42 (SD 0.90) DSM-IV Axis 1 Disorders according to the screener. At session 12, they met criteria for an average of 0.42 (SD 0.79) disorders (*t*[1.11] = 3.633, *P*=.004). At session 1, 8 out of these 12 users met screening criteria for panic disorder with agoraphobia; at session 12, only 2 continued to meet screening criteria for the disorder. In addition, 3 out of these 12 users met screening criteria for social anxiety at session 1, whereas only one met screening criteria at session 12.

At registration and at the end of session 12, users were asked a number of questions, including a question about the degree to which their panic attacks interfered with their normal daily lives on a 0 to 4 scale with 0 being *none/no interference* and 4 being *extreme/severe interference*. At registration, the average interference rating was 2.58 (SD 1.08), as compared to 0.42 (SD 0.77) at the end of treatment (*df*=1,11, *t* = 5.348, *P*<.001). At the end of session 12 users were also asked to rate the degree to which their fear/and or avoidance interfered with their normal daily life, with 0 being *none/no interference* and 4 meaning *extreme/severe interference*. On average, the 12 users who completed the survey rated this question as 0.42 (SD = 0.90).

In response to the survey at the end of session 12, 12 out of 12 (100.00%) users reported that since challenging the Panic Program they were challenging their anxious thoughts, 11 out of 12 (91.67%) reported that they were getting better at setting goals and designing exposure plans, 12 out of 12 (100.00%) reported that since starting the Panic Program they had gained confidence in their ability to challenge their fears and win, and 12 out of 12 (100.00%) reported that they believed that their hard work was paying off. Out of 12 users, 10 (83.33%) reported that they used the Support Group and 10 out of 10 (100%) rated the Support Group as “extremely helpful.”

## Discussion

### Principal Findings

This study evaluated the patterns of use and effectiveness of a Web-based self-help program for panic disorder and agoraphobia. We found that the website is popular and well utilized. Users tend to visit the website several times and spend considerable time on the website. With respect to the goal of increasing collaborative disease management and promoting communication between consumers and health care professionals, it would appear that the website is being used for that purpose. For example, approximately 50% of users who complete the WB-DAT report that they either intend to share the results with a health care professional or are considering doing so, and approximately 10% of users reported that they were using the CBT program in collaboration with a health care professional. A small but noteworthy percentage of people who registered to use the WB-DAT and CBT program, and those who downloaded the print version of the CBT program identified themselves as health care professionals.

Among the interesting findings from this study is the fact that a fairly high proportion of users who completed the WB-DAT met criteria for one or more anxiety disorders. It appears that users of the website are likely people who are self-selected because they are suffering from some type of anxiety disorder and perhaps especially panic disorder or agoraphobia. It is also interesting that most support group users were passive visitors and viewers as opposed to users who post information.

The data regarding the usage and effectiveness of the CBT program are also interesting. Although many people used the program for a few weeks, only a few used it for the entire 12 sessions. However, consistent with the literature [7-11,27,28] it appears that the CBT program can be effective in reducing panic attack frequency and severity. At the end of session 12 the remaining users reported a significant reduction in the number and severity of panic attacks and interference in daily life due to panic attacks. More importantly, the CBT program appears to have been of benefit to many users even if they used it only for a few weeks. Psychoeducation and information about anxiety, panic and avoidance may be all that many people need to feel “better enough.” In addition, there appears to be a dose-response effect between treatment duration and the degree of reduction in number and severity of panic attacks (Tables 4 and 5).

### Limitations

It is important to note that these data were collected in an uncontrolled fashion. In contrast to previous reports of controlled trials of computer and Web-based interventions, we analyzed cumulative anonymous data from a freely available program. In addition, the sample was not demographically well characterized. In order to ensure anonymity, only minimal demographic data were collected. Because this was a longitudinal design with no control group we do not know whether the highly self-selected group of users who stayed in the program would have become better also without the intervention.

The most notable problem is the high attrition rate, which is consistent with other research on self-help interventions [9,10]. For most people it is difficult to do exposure-based treatment without professional assistance [11,12].

The high attrition rate may also be caused by the option of downloading a PDF file of the entire Panic Program. Given that 1161 users registered for the program and 1159 users downloaded the PDF version, it seems likely that many users preferred to read from the hard copy. They may have stopped using the Web-based program and their data regarding their usage of the program was therefore lost. However, they may have continued to use the hard copy to some effect. It also may be that many people choose to use self-help resources in a nonlinear manner.

### Comparisons with Other Studies

The results of this study are consistent with the results of recent research demonstrating the efficacy of Web-based self-help for panic disorder [27], the efficacy of freely available Web-based self-help programs for mood and anxiety problems [28], and the high attrition rates reported in other studies of self-help interventions [9,10].

### Summary and Questions to be Addressed by Further Research

In summary, despite the high attrition rate, these data suggest that freely available Web-based self-help for panic disorder can be effective for self-selected individuals. Such a result is interesting given the cost-effectiveness of Web-based treatments compared to conventional psychotherapeutic treatment and the potential for Web-based interventions to reach people in need [12, 38,39]. It seems likely that attrition rates can be reduced by making Web-based self-help interventions a part of a stepped model of care that includes the option of some minimal amount of therapist contact and guidance. An important focus of future research will be to conduct “dose finding” studies to determine the optimal level of professional guidance and support that will facilitate treatment adherence and effectiveness for users of free Web-based programs.

## Multimedia Appendix

Additional Screenshots

## Abbreviations

CBT: Cognitive behaviour therapy
MDD: Major Depressive Disorder
WB-DAT: Web-based depression and anxiety test
