# Supplementary material for: Usage and Longitudinal Effectiveness of a Web-Based Self-Help Cognitive Behavioral Therapy Program for Panic Disorder
Source: J Med Internet Res. 2005 Mar 26;7(1):e7. doi: 10.2196/jmir.7.1.e7 (PMC1550639; doi:10.2196/jmir.7.1.e7)
Supplement: Supplementary file 1 [file jmir_v7i1e7_app1.ppt]

## Slide 1
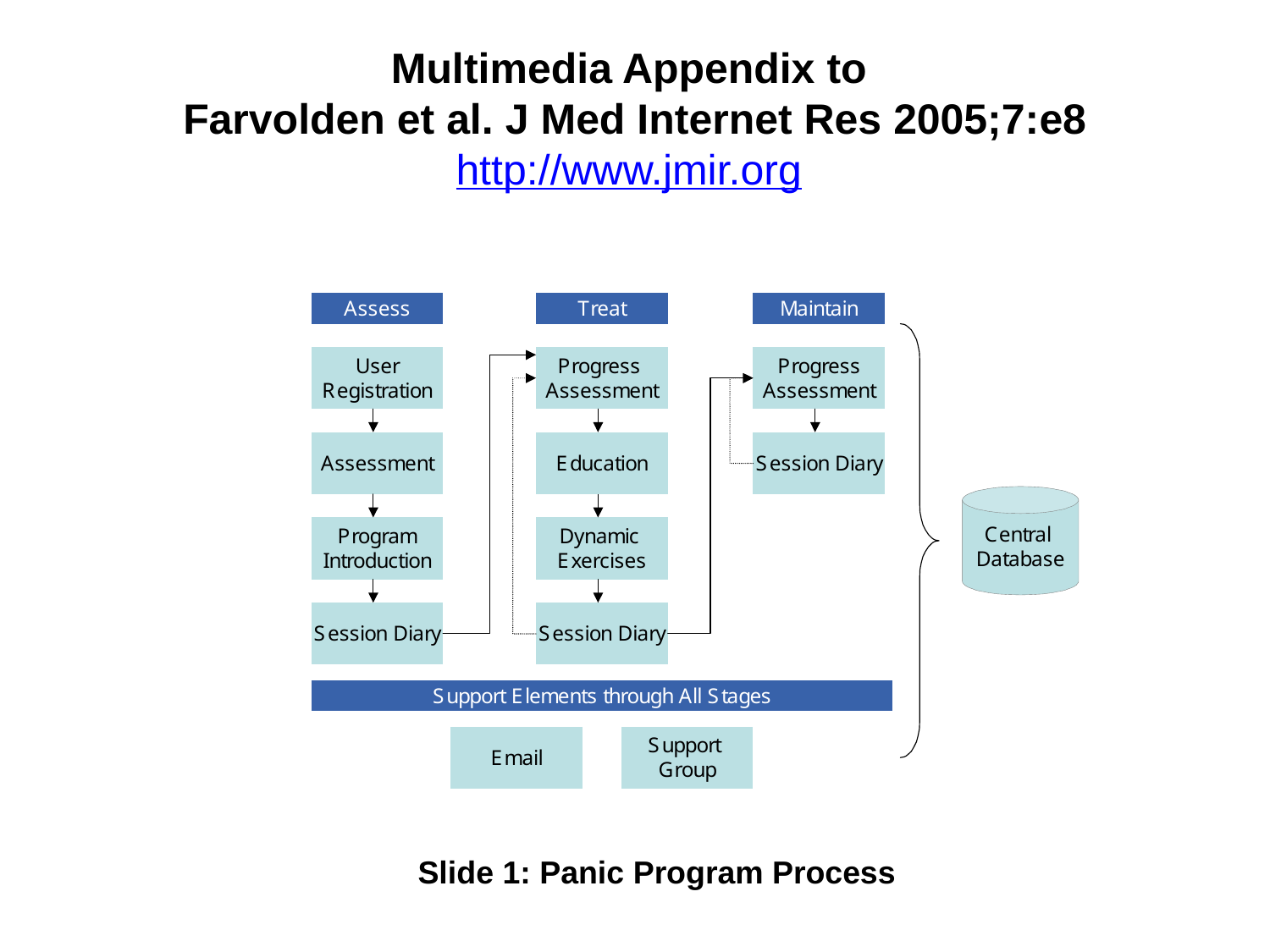

# Multimedia Appendix to Farvolden et al. J Med Internet Res 2005;7:e8 http://www.jmir.org
Slide 1: Panic Program Process

## Slide 2
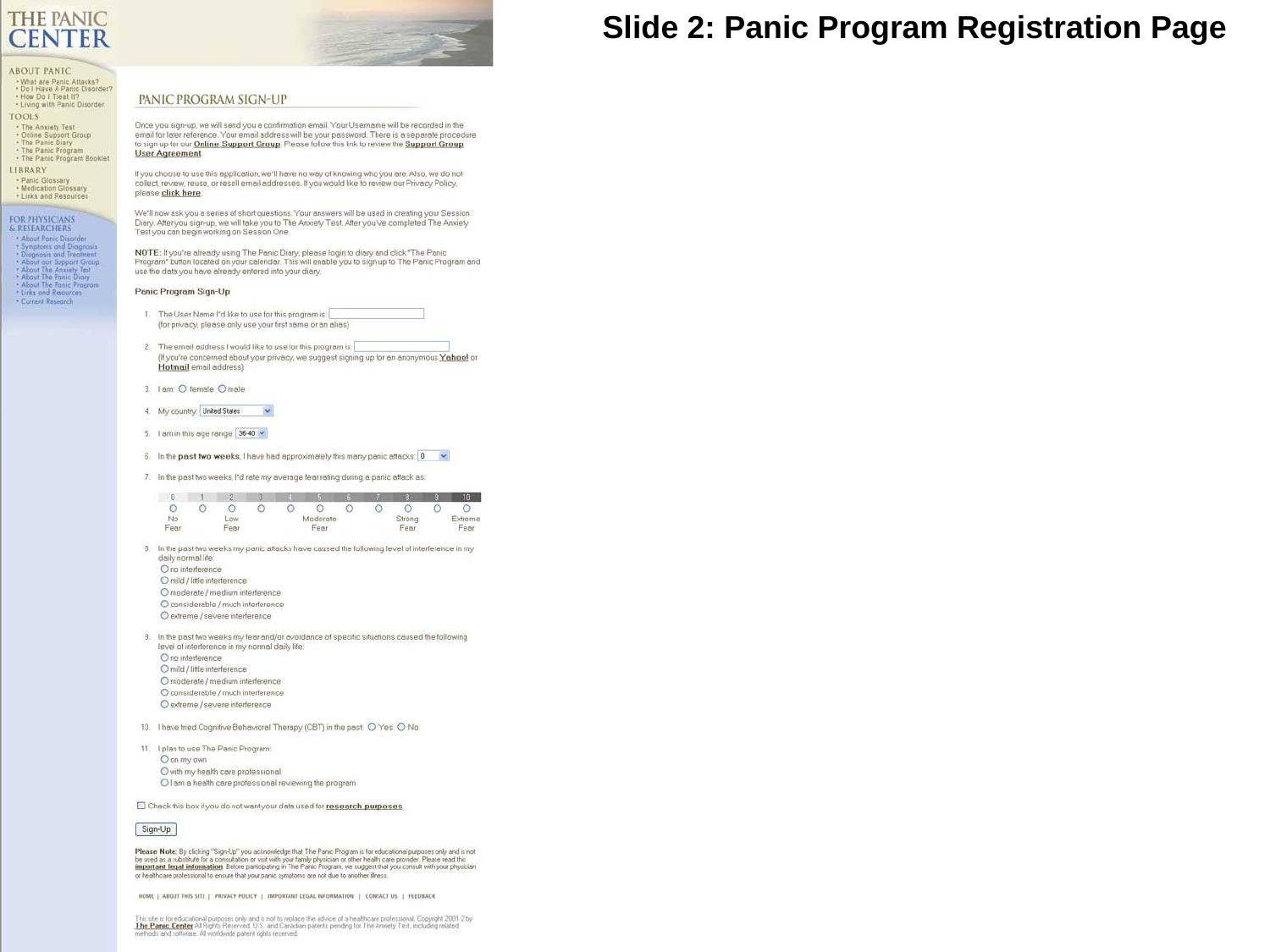

Slide 2: Panic Program Registration Page

## Slide 3
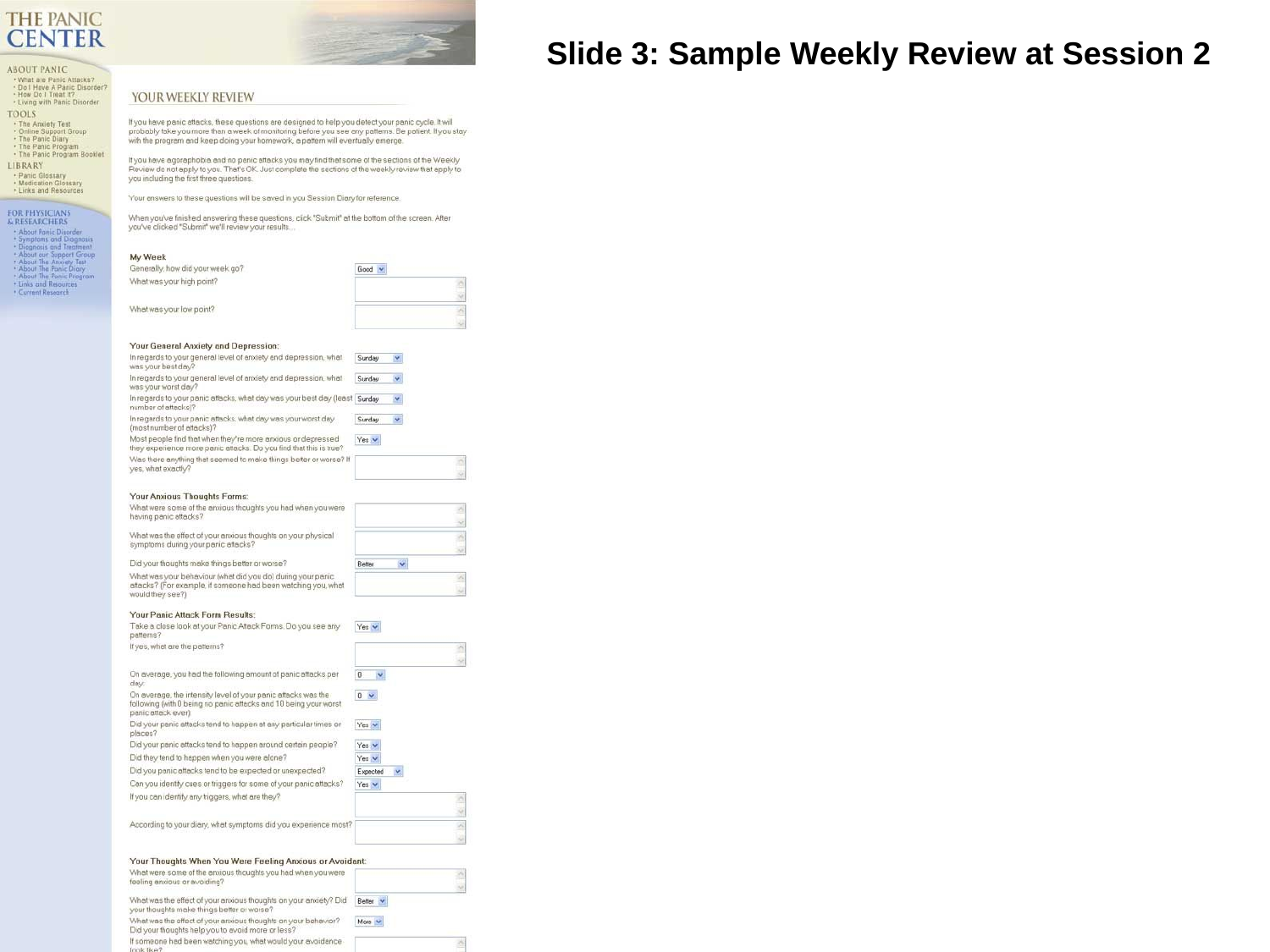

Slide 3: Sample Weekly Review at Session 2

## Slide 4
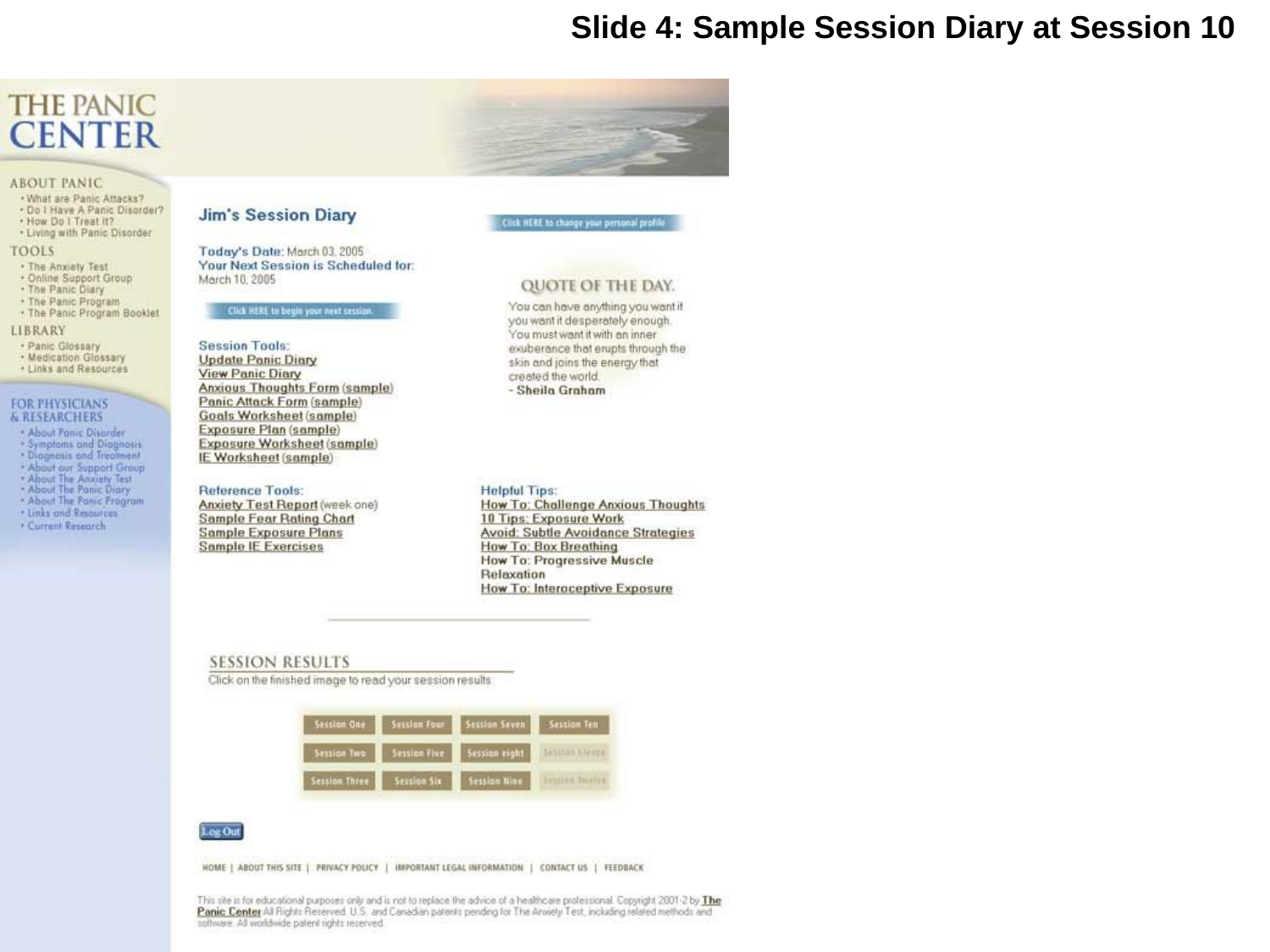

Slide 4: Sample Session Diary at Session 10

## Slide 5
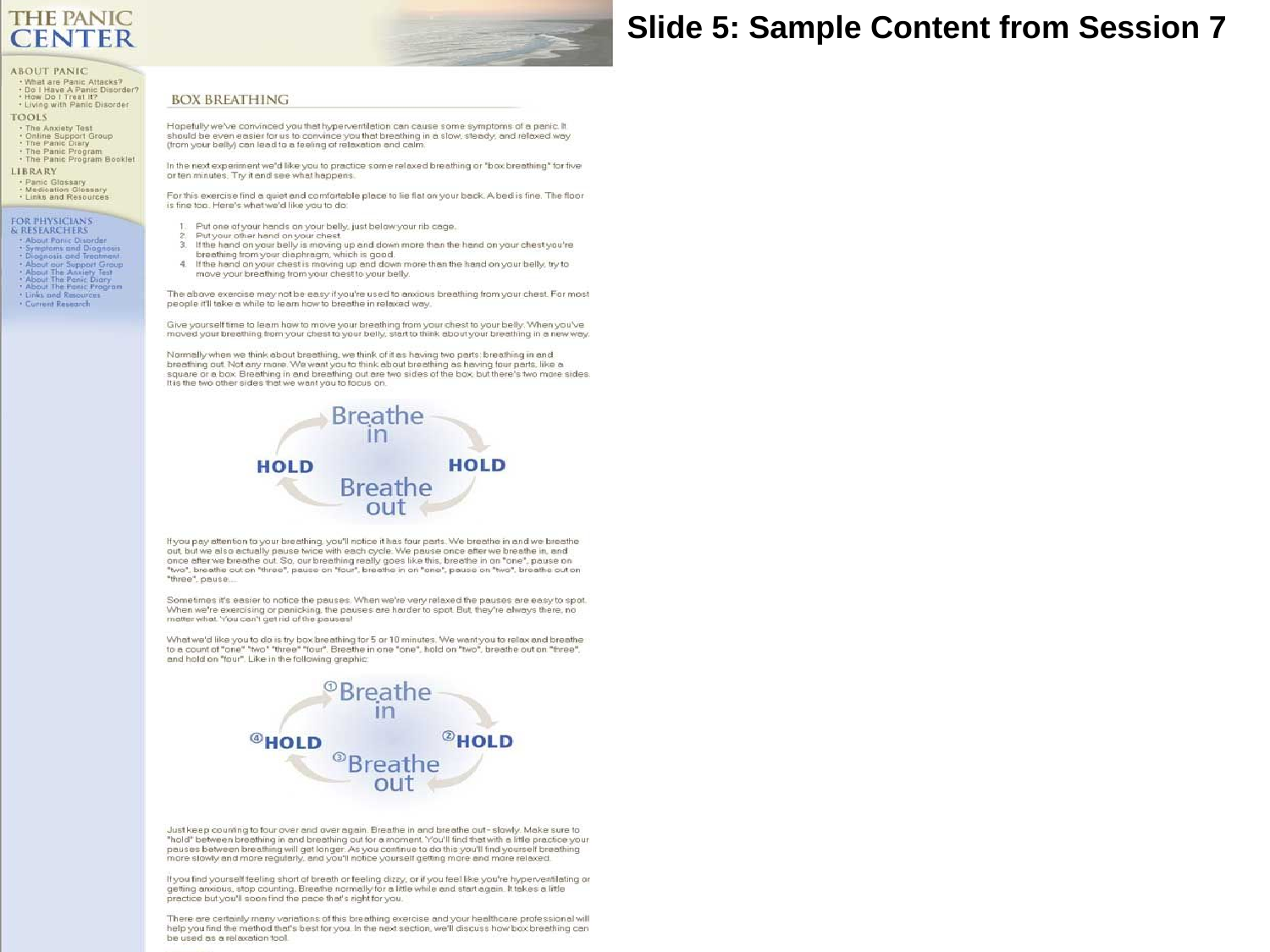

Slide 5: Sample Content from Session 7

## Slide 6
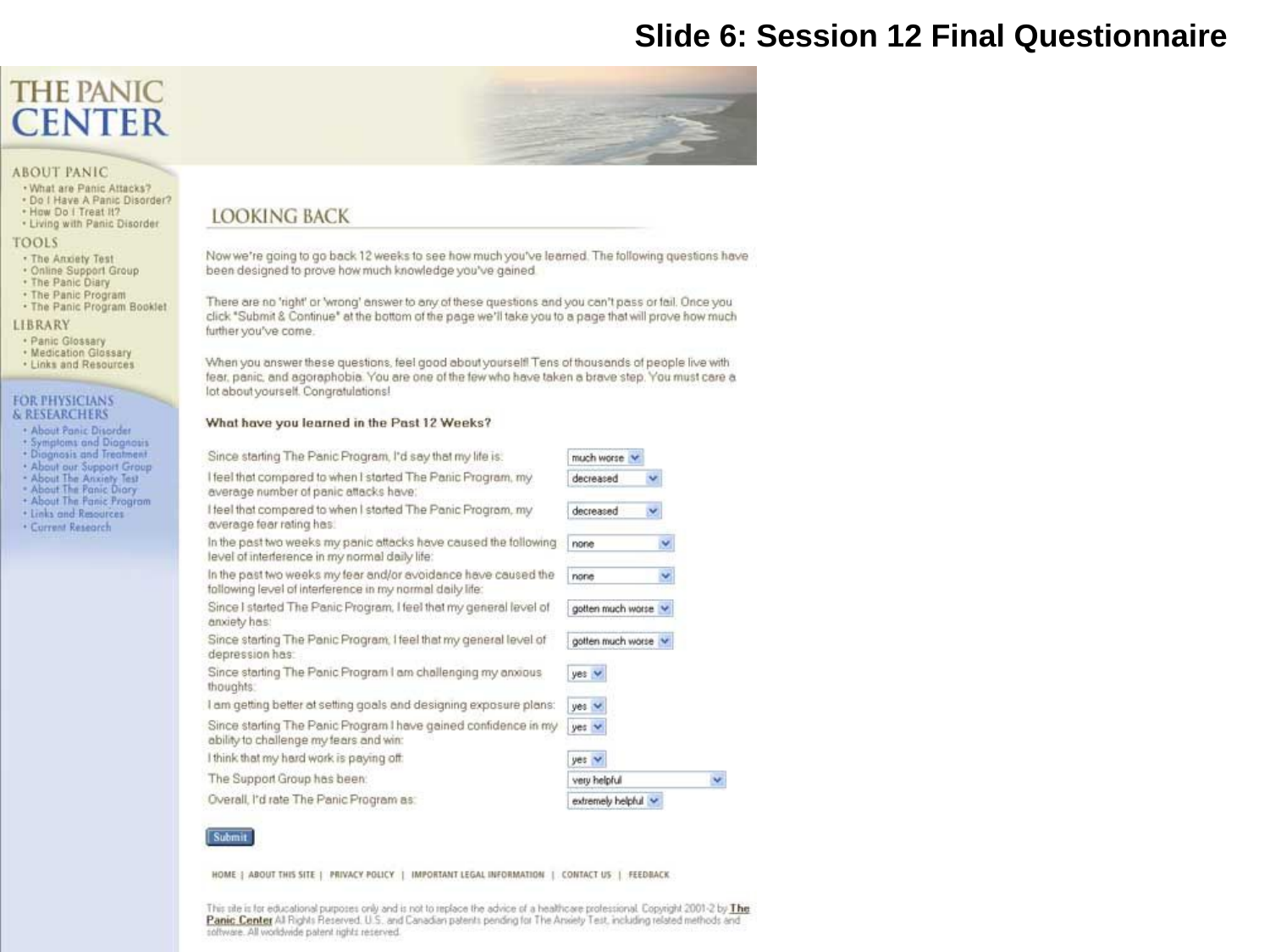

Slide 6: Session 12 Final Questionnaire

## Slide 7
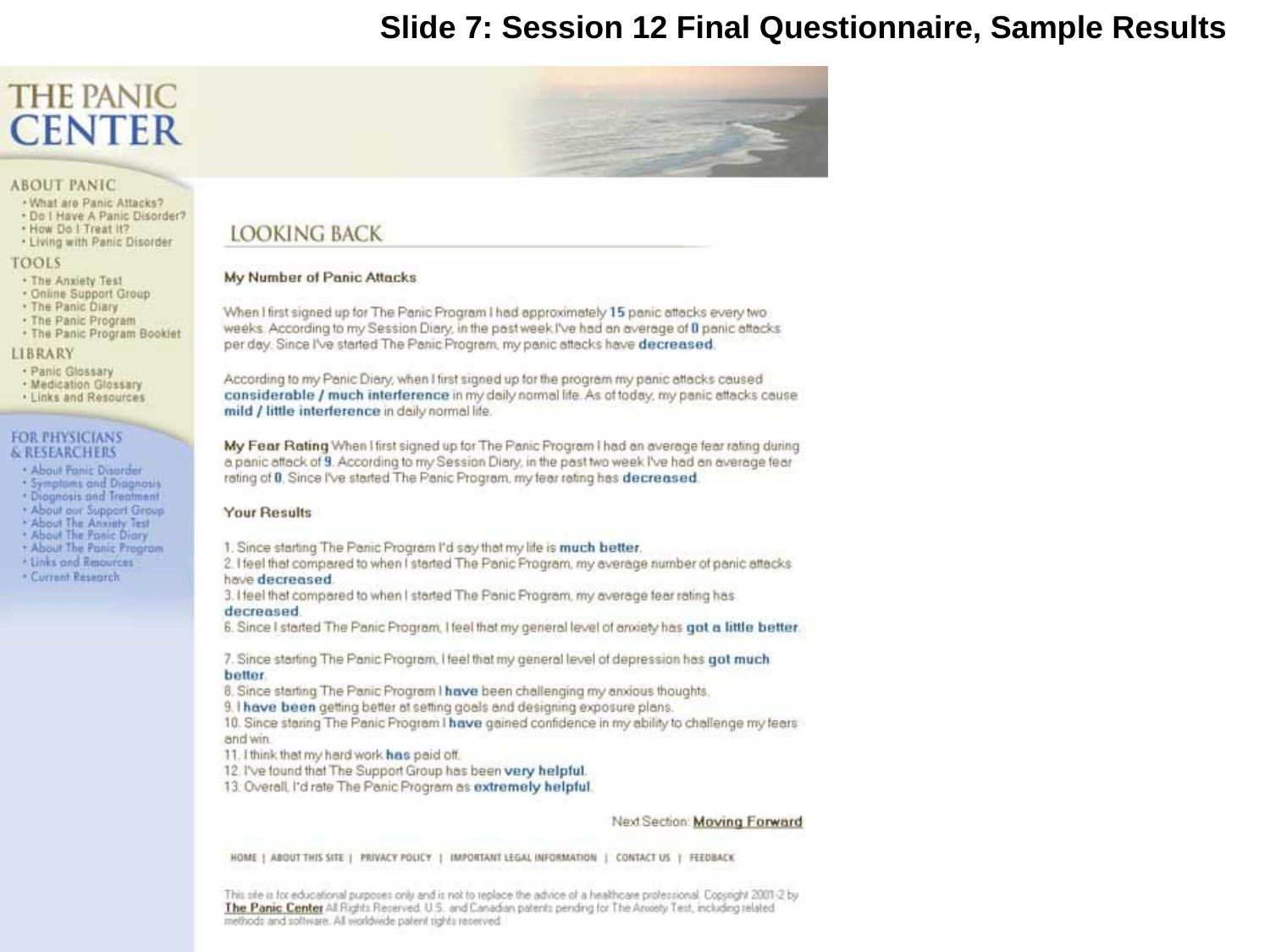

Slide 7: Session 12 Final Questionnaire, Sample Results

## Slide 8
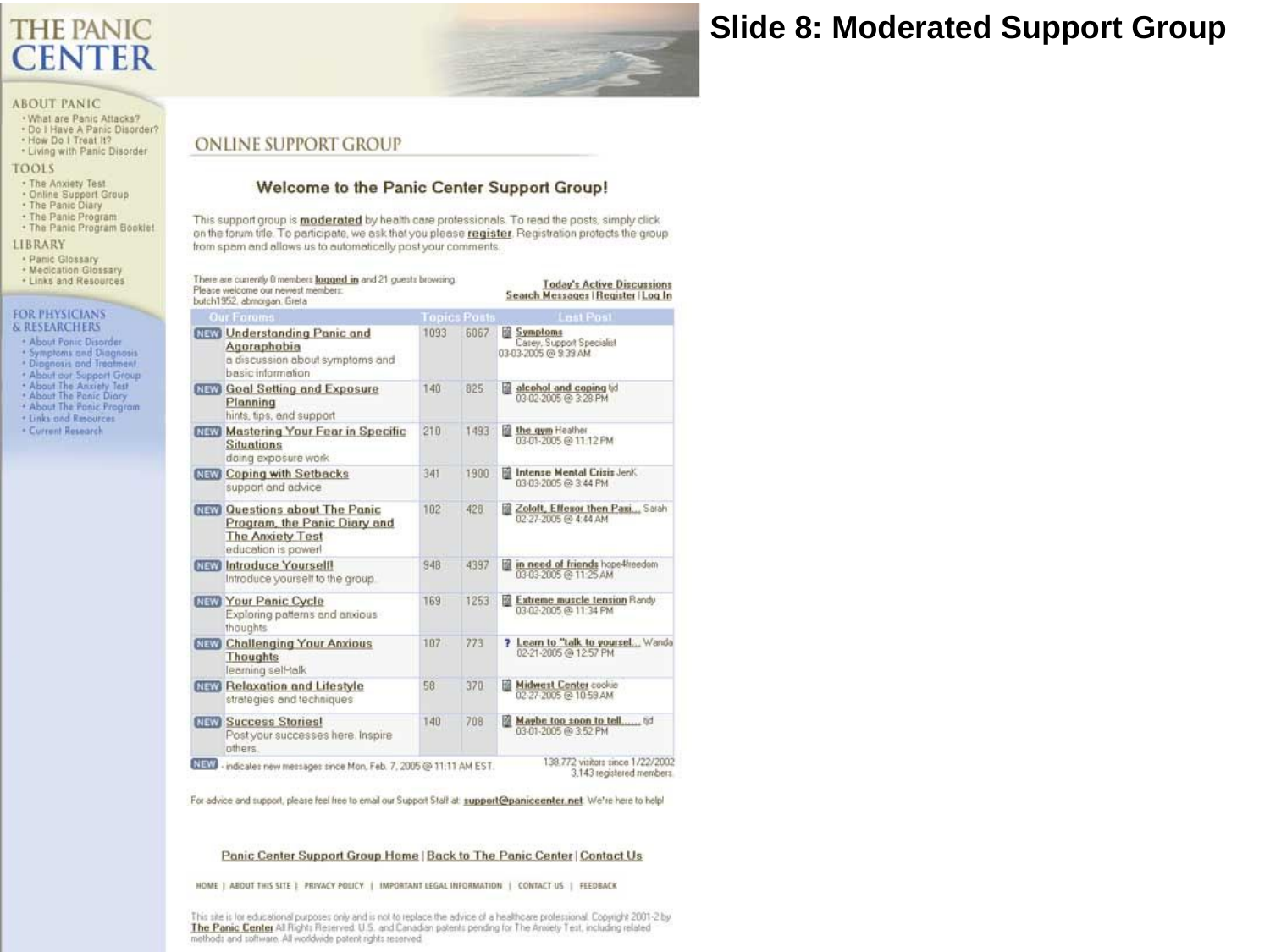

Slide 8: Moderated Support Group

## Slide 9
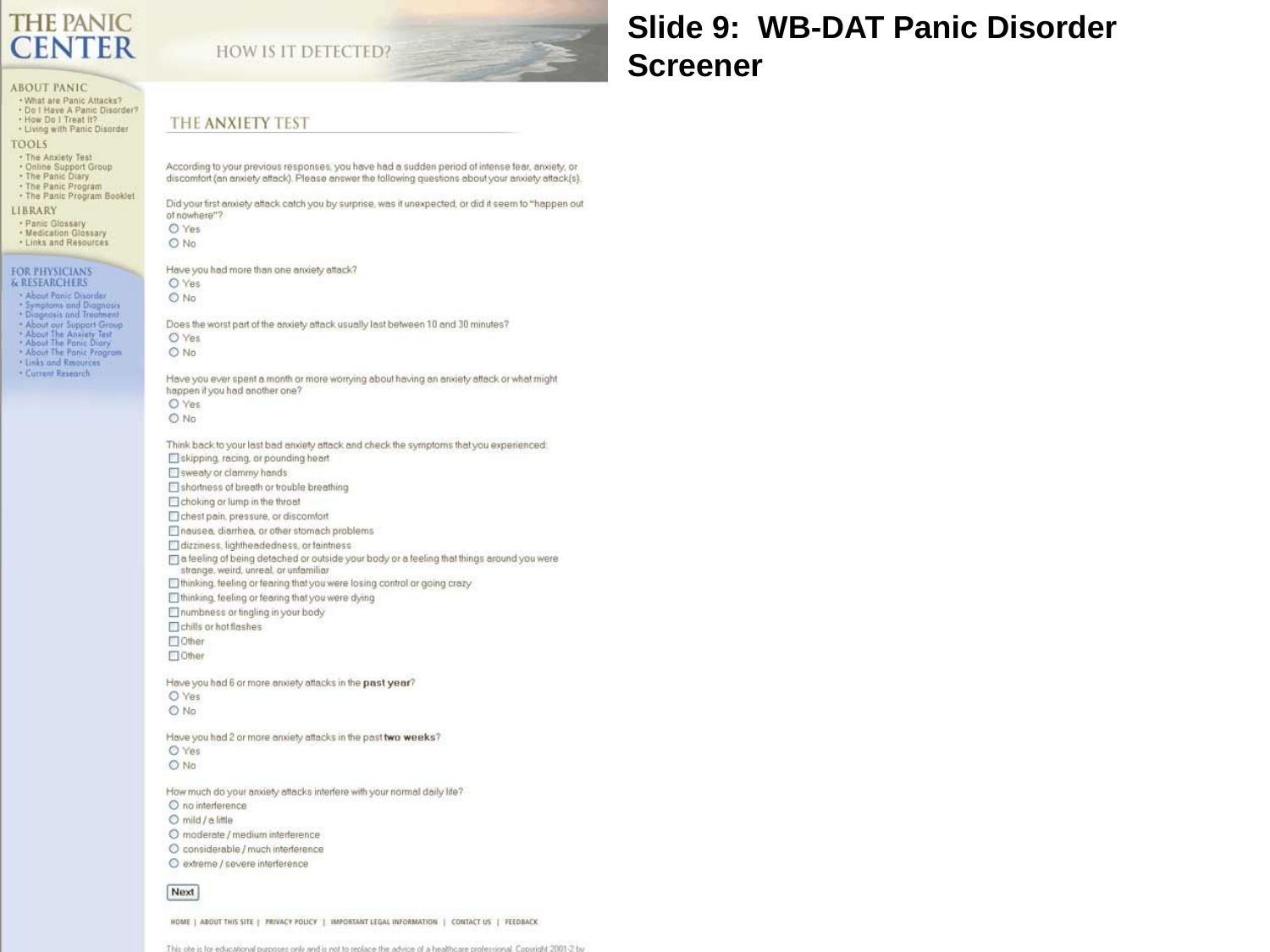

Slide 9: WB-DAT Panic Disorder Screener

## Slide 10
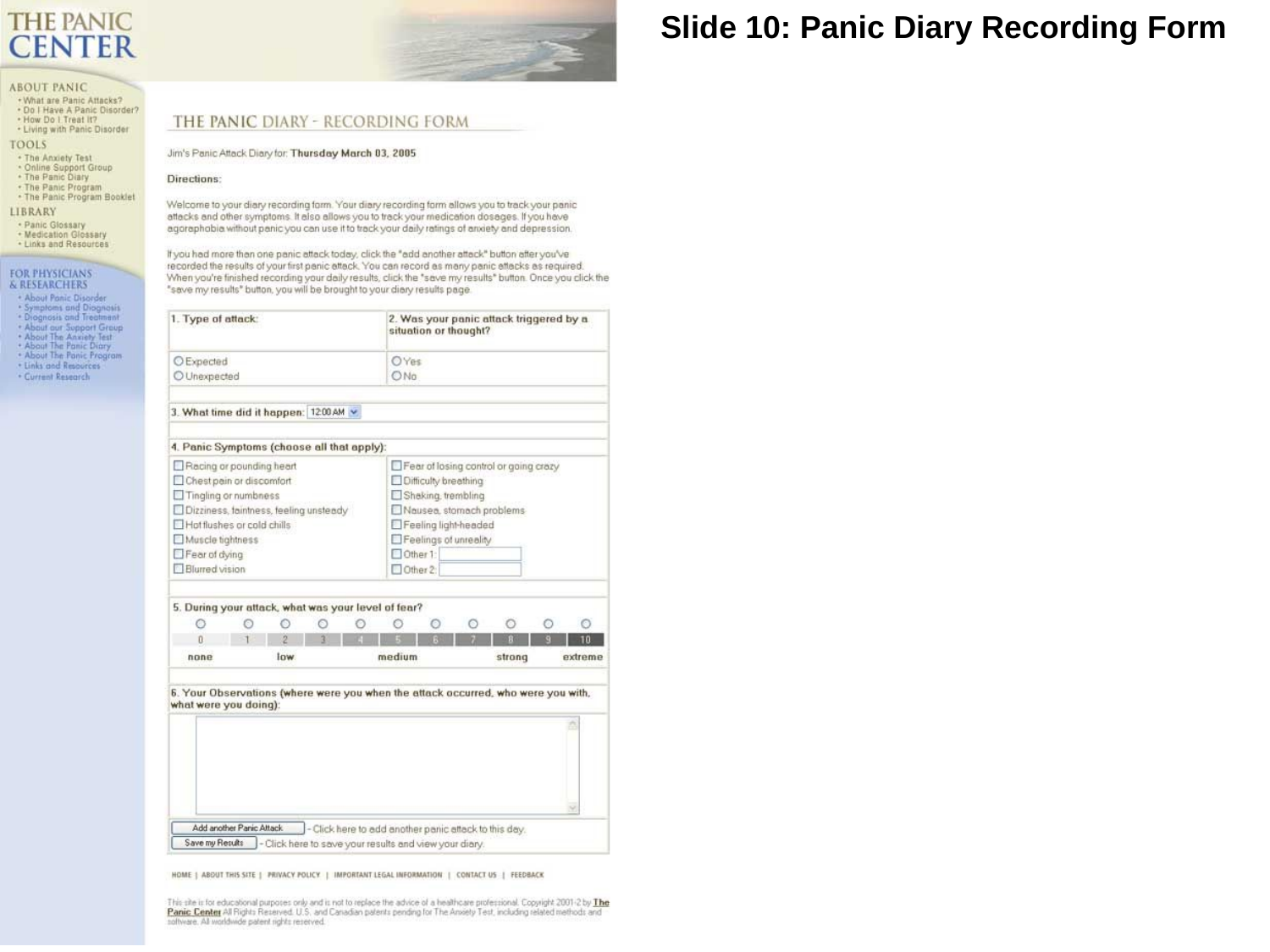

Slide 10: Panic Diary Recording Form
